# Evaluation of the antioxidant activity of *Betula pendula* leaves extract and its effects on model foods

**DOI:** 10.1080/13880209.2017.1282528

**Published:** 2017-02-02

**Authors:** Nurul Aini Mohd Azman, Monika Skowyra, Kwestan Muhammad, María Gabriela Gallego, Maria Pilar Almajano

**Affiliations:** a Chemical Engineering Department, Technical University of Catalonia, Barcelona, Spain;; b Chemical and Natural Resources Engineering Department, University Malaysia Pahang, Pahang, Malaysia

**Keywords:** Polyphenol, lipid oxidation, active packaging

## Abstract

**Context:** *Betula pendula* Roth (Betulaceae) exhibits many pharmacological activities in humans including anticancer, antibacterial, and antiviral effects. However, the antioxidant activity of BP towards lipid degradation has not been fully determined.

**Objective:** The BP ethanol and methanol extracts were evaluated to determine antioxidant activity by an *in vitro* method and lyophilized extract of BP was added to beef patties to study oxidative stability.

**Materials and methods:** Antioxidant activities of extracts of BP were determined by measuring scavenging radical activity against methoxy radical generated by Fenton reaction 2,2′-azino-bis-3-ethylbenzothiazoline-6-sulphonic acid (TEAC) radical cation, the oxygen radical absorbance capacity (ORAC) and the ferric reducing antioxidant power (FRAP) assays. The lipid deterioration in beef patties containing 0.1% and 0.3% (w/w) of lyophilized extract of BP stored in 80:20 (v/v) O_2_:CO_2_ modified atmosphere (MAP) at 4 °C for 10 days was determined using thiobarbituric acid reacting substances (TBARS), % metmyoglobin and colour value.

**Results:** The BP methanol extract revealed the presence of catechin, myricetin, quercetin, naringenin, and *p*-coumaric acid. The BP ethanol (50% w/w) extract showed scavenging activity in TEAC, ORAC and FRAP assays with values of 1.45, 2.81, 1.52 mmol Trolox equivalents (TE)/g DW, respectively. Reductions in lipid oxidation were found in samples treated with lyophilized BP extract (0.1% and 0.3% w/w) as manifested by the changes of colour and metmyoglobin concentration. A preliminary study film with BP showed retard degradation of lipid in muscle food.

**Conclusion:** The present results indicated that the BP extracts can be used as natural food antioxidants.

## Introduction

Nowadays, consumers are increasingly interested in natural foods, a healthy diet, and concern about possible toxicological effects of the synthetic antioxidants used in the food industry. Natural antioxidants owe their activity mainly from polyphenol compounds found in most herbal plants, fruits, and vegetables. Previous studies indicate that consumption of plant foods rich in antioxidants is beneficial to health and helps to prevent many diseases such as heart problems, diabetes, neurodegenerative disorder and cancers (Halliwell 1996; Decker et al. 2005; Pandey & Rizvi 2009; Aliakbarlu & Tajik 2012). Moreover, the use of natural antioxidants to replace synthetic antioxidant in model foods such as meat burgers and mayonnaise has been studied extensively in recent years. Incorporating natural antioxidants in muscle foods not only prolonged the shelf life of meat and successfully delayed the oxidation process but it also improved the nutritional quality of the meat (Sanchez-Escalante et al. 2003; Hayes et al. 2010; Doménech-Asensi et al. 2013).

Recent strategy has focused on the development of active packaging systems based on the incorporation of natural antioxidants into food packaging formulations (Gómez-Estaca et al. 2007; Bao et al. 2009). This eco-friendly biodegradable packaging not only provides safety benefits to the food, successfully controls food quality and extends the shelf life, but also replaces commercial non degradable plastic which is harmful to the environment. Biopolymers used in film preparation are derived from protein sources which have many advantages and are abundantly available. Gelatin, which is obtained by collagen hydrolysis, is one of the popular ingredients in film making and its advantages include film forming ability (Ali et al. 2014).

To date, biodegradable packaging with a natural antioxidant coating has attracted great attention, and numerous research projects are under way in this field (Bao et al. 2009; Ahmad et al. 2012; Bitencourt et al. 2014). Natural antioxidants from plants chosen for incorporation into the film contain an abundance of polyphenol constituents. Polyphenols have a wide range of beneficial health effects and their potential for delaying and inhibiting lipid oxidation has been well studied. A gelatin based film coated with 25% (w/w) lemongrass essential oil enhanced the quality and extended the shelf life of sea bass slices (Ahmad et al. 2012). The incorporation of tea polyphenol-loaded chitosan nanoparticles (TPCN) into a gelatin film improved its antioxidant activity, whereas phenolic compounds from *Curcuma longa* Linn (Rutaceae) rhizomes extract conferred barrier properties and antioxidant capacity to gelatin-based films (Bao et al. 2009; Bitencourt et al. 2014).

*Betula pendula* Roth (Betulaceae) is well known as a birch tree widely distributed in the northern hemisphere from Canada to Japan. Birch, in particular, silver birch, has traditionally been important in many countries where all of the plant parts have been utilized for various medicinal purposes. *Betula pendula* (BP) has been used to treat skin diseases, especially eczema, infections, inflammations, rheumatism and urinary disorders, and the bud oil is also widely used as a tonic and as an antiseptic in cosmetic products, particularly in hair products (Jäger et al. 2008; Jine et al. 2012; Rosado et al. 2012; Meyer-Hoffert & Brasch 2013). Furthermore, over the years there have been numerous investigations of the benefits of plants for human health (EMA: The European Medicines Agency 2014). Başer and Demirci (2007) demonstrated the potential of a few *Betula* species with antifungal, antibacterial and antioxidant effects using various *in vitro* techniques. It has been reported by Germanò et al. (2012) that the BP extract contained many polyphenol constituents such as catechin, *p*-coumaric acid, myricetin, quercetin and kaempferol, which are known for their free radical scavenging and antioxidant properties. This high content of phenolic compounds may make *Betula* species suitable for use as antioxidant sources in the food industry.

However, the antioxidant activity of BP leaf extract towards lipid oxidation has not been fully determined yet. Thus, our goals were (1) to identify the phenolic compounds in a BP extract that contribute to the antioxidant activity in the plant using LCMS, (2) to evaluate the antioxidant activity of BP using *in vitro* assays including FRAP, TEAC, ORAC and EPR scavenging activity and (3) to demonstrate the ability of BP extract to inhibit lipid deterioration in beef meat, either by inclusion in the patty composition or in the formulation of the active packaging.

## Materials and methods

### Materials

BP leaves were purchased from Pàmies Hortícoles (Balaguer, Spain) in spring 2014 and confirmed taxonomically by Josep Pàmies, the owner of the registered herbal company. *Betula pendula* is listed in the herbarium of the University of Navarra (Pamp 3045).

All reagents and solvents used were of analytical grade and obtained from Panreac (Barcelona, Spain) and Sigma Aldrich (Gillingham, England).

### Extraction of *Betula pendula*

The preparation, extraction and recovery of BP was carried out according to the method of Azman et al. (2015). Dried leaves of BP were finely ground using a standard kitchen food processor. Ground BP was extracted in three different ways: (1) with 50:50 (v/v) ethanol:water; (2) with 75:25 (v/v) ethanol:water and (3) with 90:10 (v/v) ethanol:water, always in the ratio 1:30 (w/v). The extractions were performed with constant stirring at 4 ± 1 °C for 24 h, in the dark. The extract solutions of BP were recovered by filtration using Whatman Filter paper, 0.45 μm (Whatman, GE Healthcare, Wauwatosa, WI). Part of the supernatant was taken for subsequent use to determine the antiradical capacity. The volume of the remaining supernatant was measured and the excess of ethanol was removed under vacuum using a rotary evaporator (Buchi Re111, Switzerland) and kept frozen at −80 °C for 24 h. All extracts were dried in a freeze dryer (Unicryo MC2L −60 °C, Germany) under vacuum at −60 °C for three days to remove moisture. Finally, lyophilized BP was weighed to determine the soluble solids concentration (g/L).

### Determination of phenolic compound using LCMS

Preparation of 50% (v/v) BP methanol extracts was carried out as described in the extraction method above. The LCMS analysis procedure was similar to that reported by Skowyra et al. (2014) with minor modifications. Compounds were determined using a LC-ESI–QTOF-MS system acquired from Agilent with a 1200 Series HPLC (Wilmington, DE). The LC instrument has two isocratic high-pressure mixing pumps, a vacuum degasser unit and a chromatographic oven. The QTOF mass spectrometer was an Agilent 6520 model, furnished with a Dual-Spray ESI source. Compounds were separated in an Agilent Zorbax Eclipse XDB C18 column (100 mm × 2 mm, 3.5 m) connected to a C18 (4 mm × 2 mm) guard cartridge from Phenomenex (Torrance, CA). Ultrapure water (A) and acetonitrile (B), both containing 0.1% formic acid, were used as mobile phases applying the following gradients: 0–10 min, 3% B; 15–17 min, 100% B; 11 min 3% B. The mobile phase flow was 0.2 mL/min, the injection volume for standards and sample extracts was 10 μL and the column temperature was set at 30 °C. The mobile phase flow was 0.2 mL/min, using the gradients: 0–10 min, 3% B; 10–25 min, 100% B; 27–38 min, 3% B. Nitrogen (99.999%), was used as nebulizing (35 psi) and drying gas (330 °C, 10 °C/min) (Carburos Metálicos, A Coruña, Spain). The QTOF instrument was operated in the 2 GHz mode (Extended Dynamic Range, mass resolution from 4500, at *m/z* 100, to 11,000, at *m/z* 900) and compounds were ionized in positive ESI, applying capillary and fragmentor voltages of 3500 and 160 V, respectively. The Mass Hunter Workstation software was used to control all the acquisition parameters of the LC-ESI-QTOF-MS system and also to process the obtained data. Full scan MS spectra were acquired in the range from 100 to 1700 *m/z* units. The identification of polyphenol composition was based on the accurate masses, isotopic abundances and spacing of signals in their cluster of ions ([M^+ ^H]^+^), obtained in the MS mode, as well as, on their MS/MS fragmentation patterns and the exact mass of product ions.

### Determination of the total phenolic content (TPC)

The Folin–Ciocalteu method was used to determine the total phenolic content (TPC) as reported by Santas et al. (2008).

### Determination of free radical scavenging activity assays

#### Antioxidant capacity determination in vitro

Three different methods were used for the evaluation of the antioxidant activity of the extracts: 2,2′-azino-bis-(3-ethylbenzthiazoline)-6-sulphonic acid (ABTS^•+^) TEAC assay (Almajano et al. 2008), Oxygen Radical Absorbance Capacity (ORAC) assay (Skowyra et al. 2014) and Ferric Reducing Antioxidant Power (FRAP) method (Gallego et al. 2013). Results were expressed as μM of Trolox equivalent (TE) per gram of dry weight of plant (DW).

#### Electron paramagnetic resonance (EPR) spectroscopy radical scavenging assay

EPR radical scavenging activity was measured following the method of Azman et al. (2014a). The extraction was executed in MeOH with 1:10 (w/v) ratio and the soluble concentration of BP was determined by lyophilization. A spin-trapping reaction mixture consisted of 100 μL of DMPO (35 mM); 50 μL of H_2_O_2_ (10 mM); 50 μL BP extract at different concentrations or 50 μL of ferulic acid used as reference (0–20 g/L) or 50 μL of pure MeOH used as a control; and, finally, 50 μL of FeSO_4_ (2 mM), added in this order. The final solutions (125 μL) were passed to a narrow (inside diameter =2 mm) quartz tube and introduced into the cavity of the EPR spectrometer. The spectrum was recorded 10 min after the addition of the FeSO_4_ solution, when the radical adduct signal is greatest. X-band EPR spectra were recorded with a Bruker EMX-Plus 10/12 spectrometer under the following conditions: microwave frequency, 9.8762 GHz; microwave power, 30.27 mW; centre field, 3522.7 G; sweep width, 100 G; receiver gain, 5.02 × 10^4^; modulation frequency, 100 kHz; modulation amplitude, 1.86 G; time constant, 40.96 ms; conversion time, 203.0 ms.

### Determination of antioxidant activity in food model

#### Preparation of beef patties

The initial preparation method of beef patties was adapted from Azman et al. (2014b). The meat was divided into 4 batches and was mixed with 1.5% NaCl (w/w) and either (i) control (no addition), (ii) 0.1% BHT, (iii) 0.1% lyophilized BP, (iv) 0.3% lyophilized BP and moulded into smaller portions (about 20 g each). The modified atmosphere was maintained by using polystyrene B5-37 (Aerpack) trays which were placed in BB4L bags (Cryovac) of low-gas permeability (8–12 cm^3^ m^2^/24 h). The air in the trays was flushed with 80:20 (v/v) O_2_:CO_2_ by EAP20 mixture (Carburos Metalicos, Barcelona) and the trays were packaged. Samples were stored in the dark at 4 ± 2 °C for 10 days to analyze the extent of oxidation by the thiobarbituric acid reactive substances (TBARS) method, % metmyoglobin, colour, pH and microbial quality. Every measurement was carried out in triplicate each day for 10 days (except for microbiological analysis which was done every 3 days).

#### Thiobarbituric acid reacting substances (TBARS)

Lipid peroxidation was taken as an indicator of oxidative damage and was assessed by measuring the content of thiobarbituric acid reactive substances (TBARS) (Grau et al. 2000). The modified method was adapted from Azman et al. (2014b). All results were reported in mg malondialdehyde per kg of sample (mg MDA/kg sample).

#### Colour measurement and metmyglobin percentage

Objective measurements of colour were performed using a CR 400 colorimeter (Minolta, Osaka, Japan). Each patty was cut and the colour of the slices was measured three times for each point. A portable colorimeter with the settings: pulsed xenon arc lamp, 0° viewing angle geometry and aperture size 8 mm was used to measure meat colour in the CIELAB space (Lightness, L*; red-ness, a*; yellowness, b*). Before each series of measurements, the instrument was calibrated using a white ceramic tile. The metmyoglobin percentage was determined by the method developed by Xu et al. (2010). All sample measurements were carried out in triplicate.

### Development of gelatin-film with antioxidant coating

The fabrication and characterization of the gelatin based film with antioxidant coating was based on the method of Bodini et al. (2013). Whilst the filmogenic solution was cooled after the solubilization of sorbitol, 0.1% (w/w) of BP extract/gelatin and 0.1% (w/w) BHT/gelatin was added. The lipid degradation was measured using the TBARS method as mentioned above.

### Statistical analysis

A one-way analysis of variance (ANOVA) was performed using Minitab 16 software program (*α* = 0.05). The results were presented as mean values (*n* ≥ 3).

## Results and discussion

### Extraction yield, total phenolic content (TPC) and antioxidant activity

A number of studies have found that the antioxidant activity of BP extracts correlates with the phenolic content, and thus the identification of the phenolic compounds in the plant extract may reveal compounds responsible for its antioxidant activity in various assays (Germanò et al. 2012; Bertrams et al. 2013; Raudonė et al. 2014). Five polyphenol constituents were found in the 50% methanol extract of BP (Table 1) which had all been reported previously (Germanò et al. 2012, 2013). Moreover, previous studies found more than 26 polyphenol constituents in the methanol extract including kaempferol and its derivatives. Studies of the *Betula* spp. constituents including BP have been reported by a few authors (Germanò et al. 2012; EMA: The European Medicines Agency 2014). The chemical composition of flavonoids as the main polyphenolic group of constituents in birch leaves has been investigated quite extensively for several years. For example, Raal et al. (2015) developed the use of phenolic compounds as chemical indicators of a few birch species while Evans (2000) identified the chemical structures of quercetin and hyperoside as the main flavonoids in a BP extract. Among the components listed above, quercetin has been reported as the main active ingredient of birch leaves and a possible synergistic action of several flavonoids and phenolic components has also been described in BP active ingredients (EMA: The European Medicines Agency 2014). Isolation of flavonoid constituents from BP was investigated due to their numerous pharmacological benefits in human health.

**Table 1.** Polyphenol composition identified in methanol extract of BP using LCMS.

No.	t_R_ (min)	Molecular formula	[M-H]^-^	Proposed compound
1	14.2	C_15_H_14_O_6_	298.0718	catechin
2	17.3	C_9_H_8_O_3_	163.0401	*p*-coumaric acid
3	29.31	C_15_H_10_O_8_	317.0303	myricetin
4	31.6	C_15_H_10_O_7_	301.0354	quercetin
5	32.8	C_15_H_12_O_5_	271.0612	naringenin

### Analysis of total polyphenols and free radical activity assays

#### Total phenolic content and *in vitro* antioxidant activity

On average, BP extraction with 50% ethanol produced greater dry weight of soluble extract than 75% and 90% ethanol. Ethanol was selected for use in the extraction solvent since the alcohol is recognized as a GRAS (Generally Recognized as Safe) material which can be used for applications in the food industry (Fernández-Agullóa et al. 2013). Ethanol is also effective in the extraction of flavonoids and their glycosides, catechols and tannins from raw plant materials. Raal et al. (2015) reported that 20% of ethanol gives the highest polyphenol yield analyzed by net area under the curve (AUC) in HPLC chromatograms.

Table 2 shows that 50% ethanol extraction gave significantly higher value of phenol content compared to 90% and 75% ethanol extraction (*p* < 0.05). Generally, BP extracted with 50% ethanol showed higher antioxidant activity values in the ORAC assays (*p* < 0.05). Fernández-Agullóa et al. (2013) demonstrated higher values of phenolic content and TEAC values of the ethanol extract than petroleum ether Soxhlet extracts. The ORAC assay gave the highest antioxidant activity values compared to the FRAP and TEAC assays, and showed the scavenging activity of the extract towards peroxy radicals (OOH^•^) generated in the assay. The literature has reported the antioxidant activity of BP analyzed using the DPPH method, nitric oxide scavenging activity and the reducing power assay. To the best of our knowledge, this is the first report of the antioxidant activity of extracts from BP assessed using the ORAC methods.

**Table 2.** Soluble concentration, polyphenol content and antioxidant activity of BP extracts.

Activity of *Betula pendula*	Extraction solvent		
	90:10 (v/v) EtOH:H_2_0	75:25 (v/v) EtOH:H_2_0	50:50 (v/v) EtOH:H_2_0
Soluble concentration (g/L)	19.83 ± 0.05^a^	20.1 ± 0.02^b^	22.6 ± 0.05^c^
Total phenolic content (mg GAE/g DW)	10.8 ± 0.05^a^	9.11 ± 0.03^b^	11.23 ± 0.02^ac^
FRAP (mmol of TE/g DW)	1.59 ± 0.02^a^	1.06 ± 0.06^b^	1.52 ± 0.01^ac^
TEAC (mmol of TE/g DW)	1.27 ± 0.05^a^	1.36 ± 0.03^ab^	1.45 ± 0.02^bc^
ORAC (mmol of TE/g DW)	1.56 ± 0.05^a^	1.67 ± 0.05^b^	2.81 ± 0.03^c^

Values are mean ± standard deviation (*n* = 3).

GAE: gallic acid equivalent; TE: trolox equivalent; DW: dry weight.

^a–c^Means within a row with different letters are significantly different (*p* < 0.05).

#### EPR scavenging radical assay

**Figure 1.**
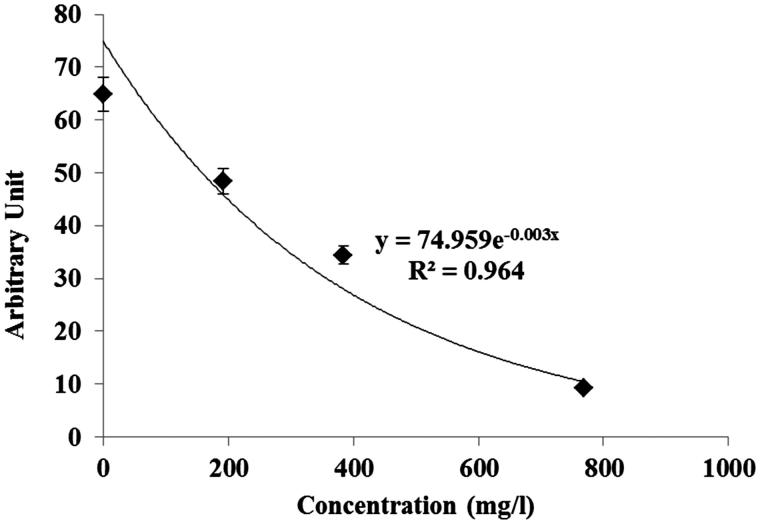
Area of the electron paramagnetic resonance (EPR) spectra of the radical adduct DMPO-OCH_3_ generated from a solution of H_2_O_2_ [2 mM] and FeSO_4_ [0.04 mM] with DMPO [14 mM] as spin trap in MeOH as solvent. The area of the EPR signal is plotted against concentration of BP methanolic extracts.

The EPR radical scavenging method has been developed by Azman et al. (2014a) to evaluate the free methoxy radical (CH_3_O^•^) generates by Fenton reaction and this was applied to the BP extract. Figure 1 showed the decreasing signal of EPR with increasing concentration of BP extract. The free radical scavenging activity of BP extracts against methoxy (CH_3_O^•^) radical was investigated by a competitive method in the presence of DMPO as spin trap, using EPR spectroscopy. The CH_3_O^•^ radical generated according to the Fenton procedure has a relatively short half-life but was identified by EPR because of its ability to form a stable nitroxide adduct with DMPO, DMPO-OCH_3_ (hyperfine splitting constants, a_N_ = 13.9 G and a_H_ = 8.3 G). This stable DMPO-OCH_3_ compound can be detected by the double integration value of the signal from the EPR spectrum. The BP extract at different concentrations competed with the spin trap DMPO in the scavenging of methoxy radicals. Thus, the antioxidant decreased the amount of radical adducts and, accordingly, decreased the intensity of the EPR signal. The best fitting with intensity of EPR signal was shown as exponential function (Figure 1) that, if concentration values are in g/L, corresponds to Equation (1):

(1) $$y=74.959e-0.003x;R^{2}=0.964$$

The graph indicates the exponential relationship of the decrease in signal in the spectrum as the concentration of BP increased. This study confirmed that the scavenging activity of the BP extracts containing polyphenol constituents could be measured by the decrease of the intensity of the spectral bands of the adduct DMPO-OCH_3_ in the EPR spectrum. The radical scavenging activity of BP has been previously determined by various methods, including the DPPH radical scavenging activity and the ferrous ion-chelating activity as reported by Germanò et al. (2013). However, this study is the first report of the measurement of BP extracts potential to act as an antiradical compound by scavenging methoxy radicals generated from the Fenton reaction.

### Antioxidant activity in food model

#### Colour and % metmyoglobin

Meat colour is one of the most important parameters that indicate meat quality. The colour values representing lightness (L*), redness (a*), and yellowness (b*) are shown in Table 3. The initial mean lightness (CIE L*) was 29.773 ± 0.866, and the control sample showed the highest value of L* at the end of 10 days storage. The L* values showed the increase in lightness of the meat due to the increased fat percentage but the redness was reduced. At the end of the storage period, samples containing 0.3% BP demonstrated the lowest value of L* while the sample containing BHT and the sample containing 0.1% BP displayed similar values. Our results were in good agreement with those of Triki et al. (2013) and Hayes et al. (2010) who showed that reduced fat in muscle food tends to reduce L* and increase a* when compared to control fat products, while redness decreased and lightness increased with storage time. Few authors have reported that lipid oxidation results in a decrease in redness (Pérez-Álvarez & Fernández-López 2009; Kim et al. 2013a, 2013b). Beef patties containing 0.3% BP maintained a more red color (a*) throughout the display, whereas the control beef patties became discoloured from 6 days onwards in the MAP. All samples showed a significant decrease of red colour after 8 days storage (*p* < 0.05).

**Table 3.** The colour values of beef patties during refrigerated storage for 10 days.

	Time of storages (days)					
Treatment	0	2	4	6	8	10
L*						
* *Control	29.77 ± 0.87^a1^	38.68 ± 1.50^a2^	29.20 ± 0.326^a1^	37.10 ± 1.23^a2^	37.23 ± 0.45^a2^	45.61 ± 2.22^a3^
* *0.1% BHT	29.77 ± 0.87^a1^	32.86 ± 1.78^b2^	30.55 ± 0.967^b3^	35.44 ± 1.06^b4^	37.99 ± 1.19^a5^	40.58 ± 0.46^b6^
* *0.1% BP	29.77 ± 0.87^a1^	34.47 ± 1.73^c2^	30.91 ± 1.518^b3^	37.30 ± 0.94^a4^	41.02 ± 1.08^b5^	40.98 ± 0.46^b5^
* *0.3% BP	29.77 ± 0.87^a1^	28.42 ± 1.58^d1^	36.45 ± 1.744^c2^	33.12 ± 1.38^c3^	36.47 ± 1.99^c2^	36.23 ± 1.90^c2^
a*						
Control	7.49 ± 0.08^a1^	6.76 ± 0.29^a2^	6.54 ± 0.328^a2^	6.26 ± 0.42^a2^	4.72 ± 0.38^a3^	0.89 ± 0.01^a4^
0.1% BHT	7.49 ± 0.08^a1^	8.18 ± 0.43^b2^	9.28 ± 0.282^b3^	7.05 ± 0.26^b1^	7.36 ± 0.41^b1^	2.86 ± 0.01^b4^
0.1% BP	7.49 ± 0.08^a1^	7.37 ± 0.23^c1^	8.74 ± 0.130^c2^	7.25 ± 0.32^b1^	5.64 ± 0.15^c3^	1.24 ± 0.04^b4^
0.3% BP	7.49 ± 0.08^a1^	9.16 ± 0.18^d2^	8.68 ± 0.340^c3^	8.38 ± 0.18^c3^	6.75 ± 0.13^d4^	2.90 ± 0.01^b5^
b*						
Control	7.42 ± 0.32^a1^	4.86 ± 0.06^a2^	7.67 ± 0.362^a1^	8.55 ± 0.09^a3^	9.95 ± 0.14^a4^	6.77 ± 0.16^a5^
0.1% BHT	7.42 ± 0.32^a1^	6.68 ± 0.01^b2^	8.40 ± 0.150^b3^	8.39 ± 0.07^a3^	8.38 ± 0.06^b3^	2.10 ± 0.44^b4^
0.1% BP	7.42 ± 0.32^a1^	6.36 ± 0.23^b2^	8.96 ± 0.526^b3^	8.41 ± 0.12^a3^	8.76 ± 0.02^b3^	2.12 ± 0.33^b4^
0.3% BP	7.42 ± 0.32^a1^	9.19 ± 0.51^c2^	10.19 ± 0.270^c3^	7.90 ± 0.18^b1^	5.00 ± 0.06^a4^	6.90 ± 0.20^a5^

All values are expressed as mean ± standard deviation of three replicates.

^a–d^Mean values in the same column (variants) with different letters are significantly different (*p* < 0.05).

^1–6^Mean values in the same line (storage time) with different numbers are significantly different (*p* < 0.05).

L*= lightness; a* = redness; b*= yellowness.

Control = beef patty without antioxidants, 0.1% BHT = beef patty with 0.1% of butylated hydroxytoluene, 0.1% BP = beef patty with 0.1% of BP extract, 0.3% BP = beef patty with 0.3% of BP extract.

Consumers scrutinize meat freshness by its visual redness and the red colour reflects the state of oxidation of the pigment in the meat (Kim et al. 2013a). At the end of storage, the a* value of the control sample was significantly lower (*p* ≤ 0.05) than that of the other samples tested. Therefore the natural plant extracts affected meat colour and was potentially useful in delaying the oxidation and discoloration of the meat product. In general, no significant differences (*p* > 0.05) were observed in the b* values of samples during storage. The present findings seem to be consistent with other research which found that yellowness in meat patties is not influenced by time of storage and packaging conditions (Kim et al. 2013b; Triki et al. 2013). Meanwhile, Muthukumar et al. (2012) and Rojas and Brewer (2007) reported that the b* value of cooked and raw patties with antioxidants showed a more gradual reduction compared to control during storage.

**Figure 2.**
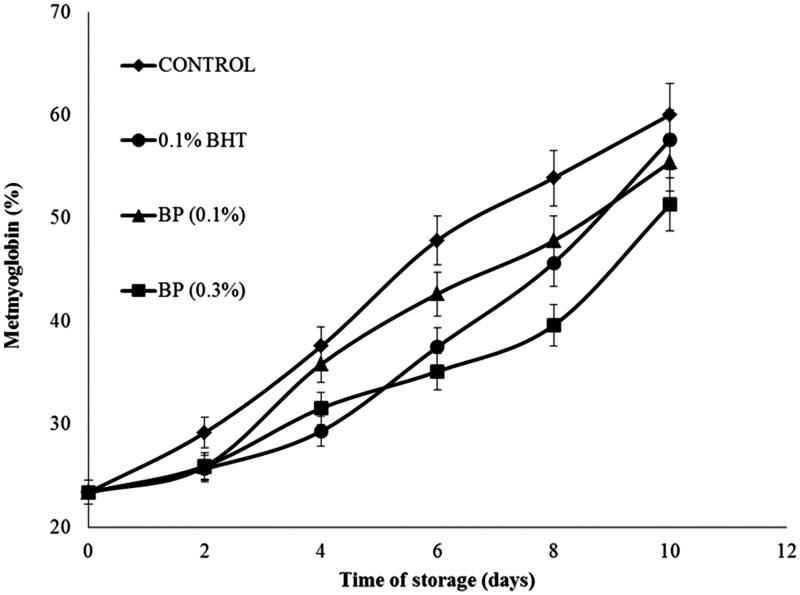
Effects of BP extract and BHT on metmyoglobin changes in beef patties during 10 days of refrigerated storage at 4 °C. Control = beef patty without antioxidants, 0.1% BHT = beef patty with 0.1% (w/w) of butylated hydroxytoluene, BP (0.1%) = beef patty with 0.1% (w/w) of BP extract, BP (0.3%) = beef patty with 0.3% (w/w) of BP extract. Each sample was measured in triplicate and the average standard deviation for each sample was less than 5%.

The changes in BP-treated samples and the effect of BHT on MMb formation during storage are presented in Figure 2. Both control and treated samples, upon storage showed an increase (*p* < 0.001) in MMb formation (*p* > 0.05). The MMb value relates to the instrumental colour a* values. Free radicals produced by lipid oxidation in meat are susceptible to initiate the reaction of oxidizing oxymyoglobin (red colour) to metmyoglobin (brown colour) which results in the discoloration of meat during storage. Samples treated with antioxidant can reduce the oxidation of metmyoglobin by scavenging hydroxyl radicals produced from oxidation of oxymyoglobin. This finding is supported by several authors who observed that the decrease of colour in muscle food is influenced by the decrease in metmyoglobin concentration during storage (Formanek et al. 2003; Xu et al. 2010).

#### TBARS analysis in beef patties

**Figure 3.**
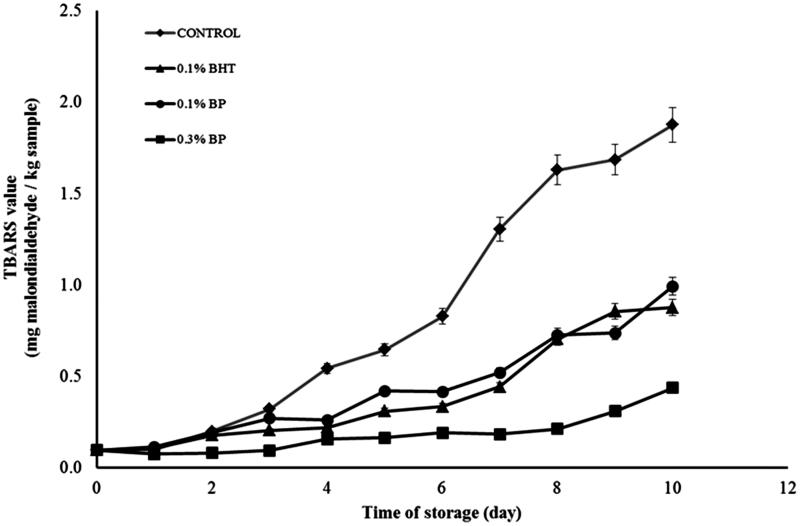
The TBARS values (mg malondialdehyde/kg sample) of beef patties in MAP atmosphere during refrigerated storage for 10 days without light. Control = beef patty without antioxidants, 0.1% BHT = beef patty with 0.1% (w/w) of butylated hydroxytoluene, 0.1% BP = beef patty with 0.1% (w/w) of BP extract, 0.3% BP = beef patty with 0.3% (w/w) of BP extract. Each sample was measured in triplicate and the average standard deviation for each sample was less than 5%.

The effect of different concentrations of BP and BHT on TBARS values in meat patties during the 10 days storage at 4 °C is shown in Figure 3. The TBARS values of all samples treated with antioxidant were significantly (*p* < 0.05) reduced compared with the control throughout the storage period in the MAP atmosphere. However, the BHT sample and 0.1% BP sample were not significantly different throughout storage. This showed that 0.1% BP would be sufficient to inhibit the generation of MDA significantly and was similar in activity to 0.1% BHT in MAP. 0.3% (w/w) BP displayed the lowest TBARS values with less than 1.0 mg malondialdehyde/kg sample at the end of the storage time; and this indicates that the sample experienced a strong antioxidant effect. The 0.1% BHT was added as comparison and is an effective dose within the legal limitation for use in food (Post et al. 2007). The effect of BP extract on lipid oxidation in meat has never been reported. Reduction of lipid oxidation with BP in meat patties in MAP could be attributed to the presence of polyphenols, rich in catechins as well as other flavonoids. The active properties of BP have been previously reported by many authors (Germanò et al. 2012; Bertrams et al. 2013; Raal et al. 2015). Our LCMS study also showed the presence of phenolic acids in the extract which may contribute to inhibition of lipid oxidation in the meat. The antioxidant activity of phenolic compounds is closely related to the hydroxyl group linked to the aromatic ring which is capable of donating hydrogen atoms and neutralizing free radicals. This mechanism blocks further degradation of active oxidized compounds such as MDA, which can be measured by the TBARS method (Kim et al. 2013a, 2013b). The study confirmed the potential of BP extract to inhibit lipid degradation in beef patties.

#### TBARS analysis in beef with active packaging

**Figure 4.**
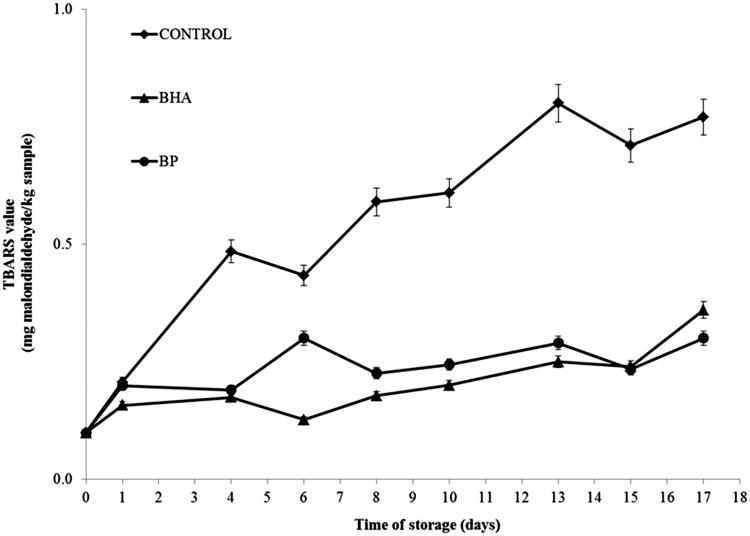
Changes in TBARS values (mg malodialdehyde/kg sample) in control and sample containing 0.1% (w/w) BHT and BP extract in MAP atmosphere during 17 days storages at 4 ± 1 °C without light. Each sample was measured in triplicate and the average standard deviation for each sample was less than 5%.

Meat packed under films coated with antioxidants, namely, BP and BHT applied at 0.1%, experienced a decrease in lipid oxidation compared to the control sample. At the first days of assay, no significant differences between the samples tested were observed. The significant differences in TBARS values occurred at day 2 (*p* < 0.05) and differences continued until 17 days storage. The samples containing synthetic and natural antioxidant showed a tendency to be marginally different (*p* < 0.1) throughout the storage period. The TBARS values of meat packed under films with 0.1% of antioxidant product also showed a significant reduction in lipid oxidation of the meat throughout the entire storage period compared to control. The increased food stability of samples containing BP and BHT can be seen in Figure 4, and it can be pointed out that the marginal difference between samples containing BP extract and BHT, which is recognized as an important antioxidant, demonstrates the effectiveness of the natural antioxidant as an alternative preservative for the food industry. The literature reports that BP contained many phenolic compounds that contribute to its strong antioxidant activity as shown by several assays (Germanò et al. 2012; Bertrams et al. 2013; EMA: The European Medicines Agency 2014).

Raudonė et al. (2014) demonstrated that the phenolic components of the BP extracts (which include hyperoside and chlorogenic acid) possess strong antioxidant activity when measured by the HPLC-FRAP post column assay. Our LCMS analysis of the BP extract showed that a few phenolic constituents such as catechin and *p*-coumaric acid exhibited antioxidant activity that has the potential to contribute to many pharmacological benefits to humans including antioxidant, and anticancer effects (Yeh & Yen 2003; Duthie et al. 2013; Kiliç & Yeşiloğlu 2013; Nithiyanantham et al. 2013). Moreover, many constituents present in the BP extract correlated significantly with antioxidant activity measured by ORAC and TEAC assays and may play important roles in the detoxification of endogenous compounds in humans (Yeh & Yen 2003).

This study was undertaken with the aim of assessing the potential effects of BP extract in delaying lipid oxidation in a food model. Beside the promising result of antioxidant activity and the presence of polyphenols in the extract, this is a preliminary study of the properties of gelatin-based film containing BP extract for environmental friendly packaging for foods. Duthie et al. (2013) demonstrated the presence of phenolic acids including *p*-coumaric acid measured using LCMS in chicken patties mixed with vegetable powders. The compound found in Duthie et al. (2013) work is relevant to our findings that the BP extract gave a good protective effect to the meat patties. Furthermore, our work also showed the efficacy of gelatin-based films treated with 0.1% BP extract.

## Conclusions

The BP extract prepared with 50% aqueous ethanol showed a high phenolic content and antioxidant activity measured by the FRAP, TEAC and ORAC assays. The various polyphenol constituents present in the BP extract contribute to the antioxidant activity and total polyphenol content. The BP extract showed scavenging ability against methoxy radicals generated by the Fenton reaction assessed by EPR. Lyophilized extracts of BP (0.1% and 0.3% w/w) can be applied as antioxidants in meat patties since these extracts inhibited lipid oxidation significantly in samples packed under MAP. 0.3% of lyophilized extract of BP significantly decreased the discoloration of meat and the brown color measured by the metmyoglobin assay. A preliminary study of the effect of gelatin-based film coated with 0.1% (w/w) BP showed that it significantly delayed degradation of lipid in meat (*p* < 0.05). Therefore, this study confirms that BP as a source of antioxidants has potential to be used by the food industry.
